# Risk for newly diagnosed diabetes after COVID-19: a systematic review and meta-analysis

**DOI:** 10.1186/s12916-022-02656-y

**Published:** 2022-11-15

**Authors:** Ting Zhang, Qimin Mei, Zhaocai Zhang, Joseph Harold Walline, Yecheng Liu, Huadong Zhu, Shuyang Zhang

**Affiliations:** 1 Emergency Department, State Key Laboratory of Complex Severe and Rare Diseases, Peking Union Medical College Hospital, Chinese Academy of Medical Science and Peking Union Medical College, Beijing, China; 2 Department of Family Medicine & Division of General Internal Medicine, Department of Medicine, Peking Union Medical College Hospital, Chinese Academy of Medical Sciences, State Key Laboratory of Complex Severe and Rare Diseases, Beijing, China; 3 Department of Critical Care Medicine, The Second Affiliated Hospital, Zhejiang University School of Medicine, Hangzhou, China; 4 Department of Emergency Medicine, Penn State Health Milton S. Hershey Medical Center and Penn State College of Medicine, Hershey, PA USA; 5 Department of Cardiology, State Key Laboratory of Complex Severe and Rare Diseases, Peking Union Medical College Hospital, Chinese Academy of Medical Science and Peking Union Medical College, Beijing, China

**Keywords:** SARS-CoV-2 virus, Diabetes mellitus, COVID-19, Unmeasured confounders

## Abstract

**Background:**

There is growing evidence that patients recovering after a severe acute respiratory syndrome coronavirus 2 (SARS-CoV-2) infection may have a variety of acute sequelae including newly diagnosed diabetes. However, the risk of diabetes in the post-acute phase is unclear. To solve this question, we aimed to determine if there was any association between status post-coronavirus disease (COVID-19) infection and a new diagnosis of diabetes.

**Methods:**

We performed a systematic review and meta-analysis of cohort studies assessing new-onset diabetes after COVID-19. PubMed, Embase, Web of Science, and Cochrane databases were all searched from inception to June 10, 2022. Three evaluators independently extracted individual study data and assessed the risk of bias. Random-effects models estimated the pooled incidence and relative risk (RR) of diabetes compared to non-COVID-19 after COVID-19.

**Results:**

Nine studies with nearly 40 million participants were included. Overall, the incidence of diabetes after COVID-19 was 15.53 (7.91–25.64) per 1000 person-years, and the relative risk of diabetes after COVID-19 infection was elevated (RR 1.62 [1.45–1.80]). The relative risk of type 1 diabetes was RR=1.48 (1.26–1.75) and type 2 diabetes was RR=1.70 (1.32–2.19), compared to non-COVID-19 patients. At all ages, there was a statistically significant positive association between infection with COVID-19 and the risk of diabetes: <18 years: RR=1.72 (1.19–2.49), ≥18 years: RR=1.63 (1.26–2.11), and >65 years: RR=1.68 (1.22–2.30). The relative risk of diabetes in different gender groups was about 2 (males: RR=2.08 [1.27–3.40]; females: RR=1.99 [1.47–2.80]). The risk of diabetes increased 1.17-fold (1.02–1.34) after COVID-19 infection compared to patients with general upper respiratory tract infections. Patients with severe COVID-19 were at higher risk (RR=1.67 [1.25–2.23]) of diabetes after COVID-19. The risk (RR=1.95 [1.85–2.06]) of diabetes was highest in the first 3 months after COVID-19. These results remained after taking confounding factors into account.

**Conclusions:**

After COVID-19, patients of all ages and genders had an elevated incidence and relative risk for a new diagnosis of diabetes. Particular attention should be paid during the first 3 months of follow-up after COVID-19 for new-onset diabetes.

**Supplementary Information:**

The online version contains supplementary material available at 10.1186/s12916-022-02656-y.

## Background

Coronavirus disease 2019 (COVID-19) is a complex clinical syndrome caused by the severe acute respiratory syndrome coronavirus 2 (SARS-CoV-2) [1]. Despite many large studies leading to the approval of vaccines and antivirals, the global spread of SARS-CoV-2 continues [2, 3]. As of June 18, 2022, there have been more than 535,863,950 confirmed cases globally, including 6,314,972 deaths (according to the World Health Organization) [4]. Factors associated with poor outcomes, including hospitalization, intensive care unit (ICU) admissions, and mortality in COVID-19 patients, are of considerable interest. More specifically, health comorbidities and baseline physical activity [5] may predispose patients to an increased risk of poor outcomes following COVID-19 infection.

Previous studies have indicated that diabetes mellitus (DM) is associated with an increased risk of severe COVID-19, acute respiratory distress syndrome (ARDS), and in-hospital mortality [6–8]. More intriguingly, a recent meta-analysis has reported that newly diagnosed diabetes is commonly observed in COVID-19 patients [9–11]. The world has raised concerns about a bi-directional relationship between these two health conditions [12].

As the COVID-19 pandemic has progressed, there is growing evidence that after the acute phase of the disease, people with COVID-19 can develop lingering sequelae (called “long COVID”) that may involve pulmonary and extrapulmonary organ system manifestations, such as diabetes [13]. Follow-up of children with COVID-19 has identified that the incidence of type 1 newly diagnosed diabetes has increased [14]. An unregistered meta-analysis [15] in PROSPERO also found an increased risk of diabetes among adults with long COVID-19, but it has some flaws in the study design which limit the interpretation and applicability of the individual studies’ findings. Therefore, there is an urgent need for systematic reviews and meta-analyses of the existing literature, particularly focusing on controlled studies.

This systematic review and meta-analysis was conducted to estimate the prevalence of a new diagnosis of diabetes after COVID-19 compared to non-COVID-19.

## Methods

This review was conducted and reported in accordance with the Meta-analysis of Observational Studies in Epidemiology (MOOSE) [16] and Preferred Reporting Items for Systematic Review and Meta-analysis (PRISMA) [17] guidelines and according to the methods described in the Cochrane Handbook for Systematic Reviews of Interventions (Additional file 2: Table S1 and Table S2). The study protocol was registered in PROSPERO on June 24, 2022 (registration number: CRD42022330723). As all included data was from previously published studies, no institutional review board approval was required.

### Search strategy and eligibility criteria

We systematically searched the following electronic bibliographic databases: PubMed, Embase, Web of Science, and the Cochrane Central Register of Controlled Trials. No time or language restrictions were applied to the search results. Full details of the search strategies used are provided in Additional file 1: Table S1. In brief, combinations of search terms were applied, relating to COVID-19 or SARS-CoV-2, diabetes mellitus, diabetes, or DM. The search was conducted from inception through June 10, 2022.

To be included in this systematic review, prospective or retrospective cohort studies had to meet all of the following criteria: (1) the main exposure of interest was COVID-19, which was defined based on International Classification of Diseases (ICD) codes; (2) to determine relative associations, hospitalized or population controls were utilized as comparators, with priority given to population controls where available; (3) report of newly diagnosed DM that was defined as the new onset of diabetes (no prior history of diabetes with a fasting plasma glucose [FPG] ≥ 7.0 mmol/L or a random blood glucose [RBG] ≥ 11.1 mmol/L or a HbA1c >6.5%). The following were excluded: case reports or case series, reviews, commentaries, and letters.

### Outcomes

The primary outcome was the incidence of newly diagnosed DM after the diagnosis of SARS-CoV-2 infection, and the relative risk of acquiring DM compared with contemporary or historical controls in the non-SARS-CoV-2 cohort. We performed subgroup analyses according to age, gender, type of DM, time of onset, and whether the control group was generalized upper respiratory tract infections. We also performed two post hoc subgroup analyses: mild-to-moderate COVID patients versus severe COVID patients, looking at three different follow-up times after COVID-19 (less than 3 months, 3 to 6 months, and greater than 6 months).

### Study selection and data extraction

Data extraction from eligible studies was performed using a standardized spreadsheet. The extracted data included items related to study design and data sources, study participant characteristics, study definition of COVID-19, definition of DM, covariates, and person-years of follow-up (either reported or calculable). The data were extracted by the first reviewer (TZ) and double-checked by a second reviewer (QMM). Disagreement between the two reviewers was resolved by discussion with a third reviewer (YCL).

### Methodological quality

The risk of bias for the included studies was assessed using the Newcastle–Ottawa quality assessment scale for cohort studies [18]. Critical appraisal was carried out by three reviewers (TZ, QMM, and YCL), with discrepancies discussed with the larger authors’ group to reach a consensus.

### Statistical analysis

For each included study, the incidence of newly diagnosed DM was calculated using the reported number of newly diagnosed diabetes cases and person-years of follow-up. We first transformed proportions using the Freeman–Tukey double arcsine method [19] and then performed an inverse variance random-effects meta-analysis (DerSimonian and Laird) [20] to calculate the pooled estimates. Diabetes rates between the SARS-CoV-2 patients and control subjects were reported using relative risk (RR) with 95% confidence intervals (CIs).

Heterogeneity between included studies was assessed with the Cochran *Q* and *I*^2^ statistics. For the qualitative interpretation of heterogeneity, *I*^2^ values of at least 50% were considered to represent substantial heterogeneity, and values of at least 75% indicated considerable heterogeneity [21]. The significance level for the *Q* statistic was set at 0.1. For outcomes reported in 10 or more studies, publication bias was explored by constructing power-enhanced funnel plots (sunset funnel plot) and an Egger’s test [22, 23].

To consider how strong uncontrolled confounders in each meta-analyzed study would have to be to negate the observed results, we applied a sensitivity analysis for meta-analyses that are analogous to the *E*-value [24, 25]. We calculated an *E*-value [26] representing the minimum strengths of associations on the risk ratio scale that uncontrolled confounders would need to jointly have with COVID-19 and with DM across all studies in each meta-analysis to shift the meta-analytic estimate or its 95% CI to the null.

All risk estimates were calculated with the corresponding 95% CIs. *p*-values <0.05 were considered statistically significant. All statistical analyses were performed with R software (version 4.1.2, Vienna, Austria) [27, 28], using the packages “metafor,” “EValue,” “confoundedmeta,” “metaviz,” and “metaUtility.”

## Results

**Fig. 1:**
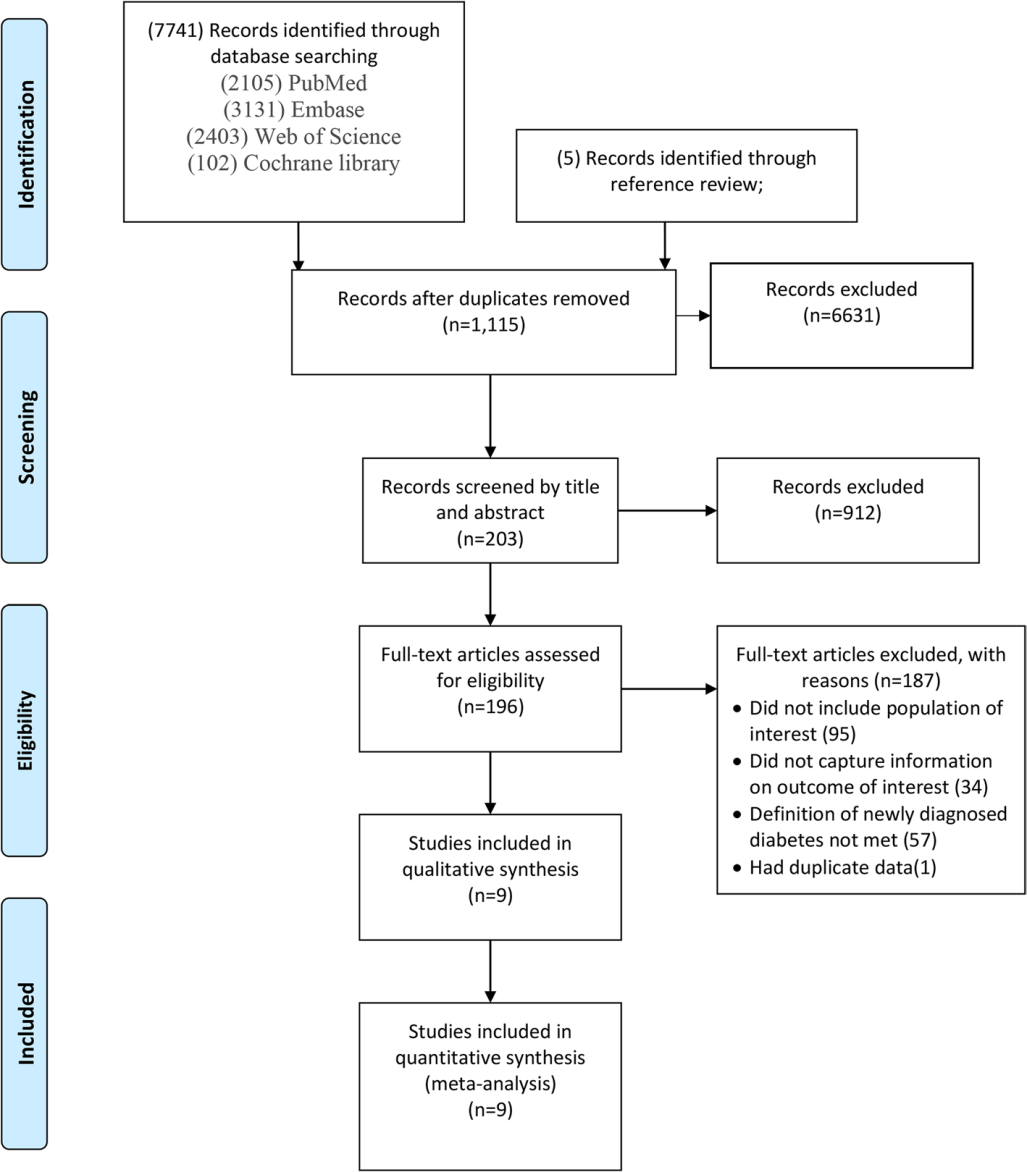
Flow chart of included studies. The number of articles which were identified, screened by abstract, screened by full text, and ultimately selected for inclusion is displayed. The numerical breakdown of the rationale for removal after the full-text review is also displayed

The search yielded 7746 citations. After duplicates were removed and titles and abstracts were reviewed, 7543 articles were excluded. Of the remaining 203 studies, full-text articles of 196 were available. Of the 196, 187 were then excluded after reviewing the full-text manuscripts. After several stages of review, nine eligible studies (with 10 cohorts) were included in the meta-analysis and eight studies were propensity score matching (PSM) cohort studies (Fig. 1) [29–37].

Table 1 and Table S2 show the characteristics of the included studies: six were conducted in the USA [29, 30, 32, 33, 35, 37], two in England [34, 36], and one in Germany [31]. Among the nine studies, seven included only adults, one study included a full population (no restrictions), and one study included only adolescents. Overall, there were 4,002,475 people diagnosed with COVID-19 and 34,717,422 people in the respective control groups.

**Table 1:** Characteristics of the total study population

First author (year)	Group	Population	Age (years), mean (SD or range), or median (IQR)	Gender (proportion)	Event number	Follow-up time	Levels of COVID-19 severity
Xie, 2022 [35]	COVID-19 group	181,280	60.92 (17.02)	Male: 159,666 (88.08%) Female: 21,614 (11.92%)	7871	352 (IQR 244–406) days	Not hospitalized; hospitalized; intensive care
	Control group	4,118,441	61.5 (17.08)	Male: 3,655,034 (88.75%) Female: 463,407 (11.25%)	127,002	352 (245–406) days	Not applicable
Rathmann, 2022 [31]	COVID-19 group	181,280	42.6 (19.1)	Male: 164,918 (54.4%) Female: 16,362 (45.6%)	241	119 (IQR 0–210)days	Mild COVID-19
	Control group	4,118,441	42.6 (19.1)	Male: 2,240,432 (54.4%) Female: 16,370 (45.6%)	228	161 (IQR 4–225)days	Not applicable
Qeadan, 2022 [33]	COVID-19 group	2,489,266	Not reported	Male: 1,081,608 (46.1%) Female: 1,264,069 (53.9%)	5163	Not reported	Unavailable
	Control group	24,803,613	Not reported	Male: 10,579,475 (45.9%) Female: 12,491,493 (54.1%)	36,348	Not reported	Not applicable
Collaborative, 2022 [34]	COVID-19 group	77,347	68	Male: 39,808 (51.5%) Female: 37,539 (48.5%)	670	274 days	Hospitalized COVID-19
	Control group	386,669	68	Male: 199,013 (51.5%) Female: 187,656 (48.5%)	2763	274 days	Not applicable
Cohen, 2022 [30]	COVID-19 group	87,337	75 (71–82)	Male: 58,110 (44%) Female: 29,227 (56%)	2463	64 (IQR 23–150) days	Not hospitalized: 63,690 Hospitalized: 23,486
	Control group	87,337	74 (70–80)	Male: 42% Female: 58%	1249	64 (IQR 23–150) days	Not applicable
Birabaharan, 2022 [32]	COVID-19 group	282,105	Not reported	Not reported	2320	180 days	Mild COVID-19: 276,748 Moderate/severe COVID-19: 5357
	Control group	286,275	Not reported	Not reported	1916	180 days	Not applicable
Barrett [IQVIA database], 2022 [29]	COVID-19 group	80,839	12.3 (4.3)	Male: 404,465 (49.9%) Female: 40,517 (50.1%)	68	362 days	Hospitalized: 6473 (0.4)
	Control group	404,465	12.3 (4.3)	Male: 58,110 (49.9%) Female: 201,880 (50.1)	132	362 days	Not applicable
Barrett [Health Verity database], 2022 [29]	COVID-19 group	439,439	12.7 (3.8)	Male: 219,427 (49.9%) Female: 220,012 (50.1%)	1120	484 days	Hospitalized: 13,118 (3.0)
	Control group	439,439	12.7 (3.8)	Male: 219,427 (49.9%) Female: 220,012 (50.1%)	853	484 days	Not applicable
Ayoubkhani, 2021 [36]	COVID-19 group	36,100	60.9 ± 17.02	Male: 54.9% Female: 45.1%	400	140 days	Intensive unit care (ICU): 4745 Non-ICU: 43,035
	Control group	36,100	61.5 ± 17.08	Male: 54.9% Female: 45.1%	125	140 days	Not applicable
Daugherty, 2021 [37]	COVID-19 group	193,113	41.7 ± 13.9	Male: 47.6% Female: 52.4%	1237	87 (IQR 45–124)days	Mild and hospitalized COVID-19
	Control group	193,113	41.6 ± 13.8	Males: 47.5% Female: 52.5%	649	87 (IQR 45–124)days	Not applicable

The risk of bias in the included cohort studies was assessed using the Newcastle–Ottawa Scale and is presented in Table S3. The overall score was 88 of 90 (97.8%), which is considered to be a relatively low risk for bias.

**Fig. 2:**
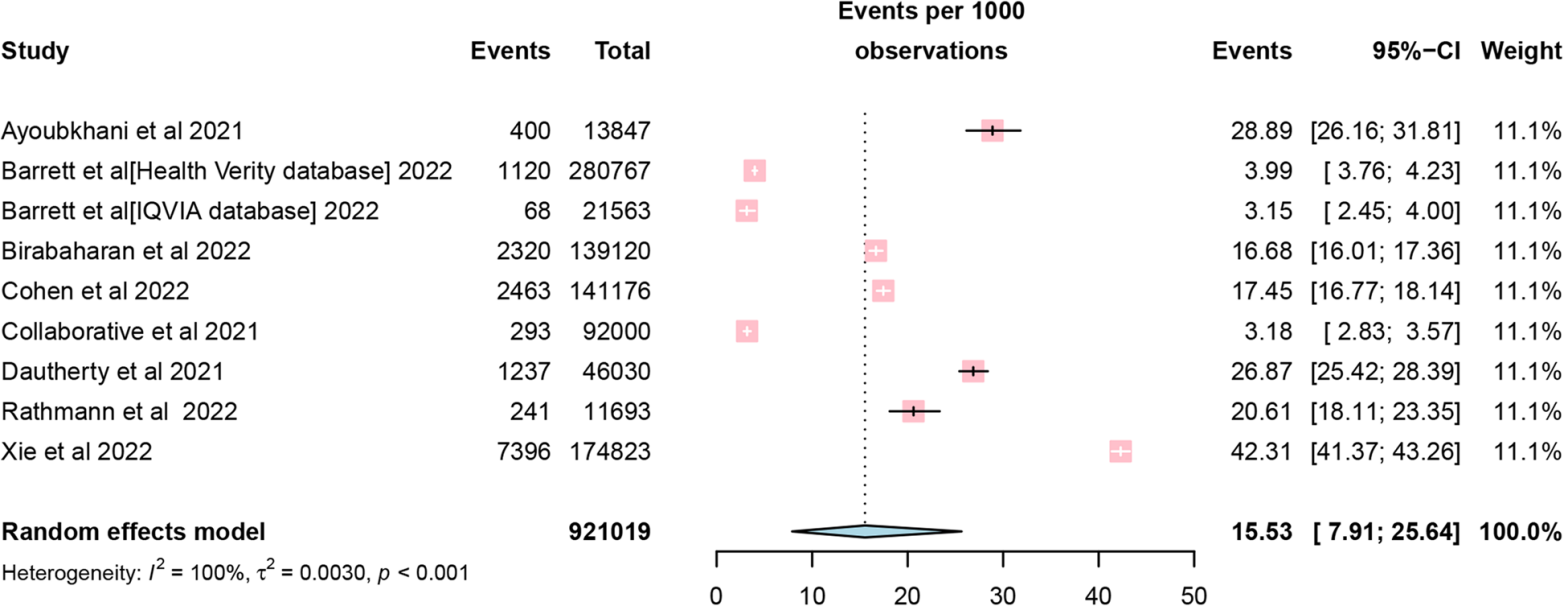
Forest plot of diabetes incidence among COVID-19 people. CI confidence interval

**Fig. 3:**
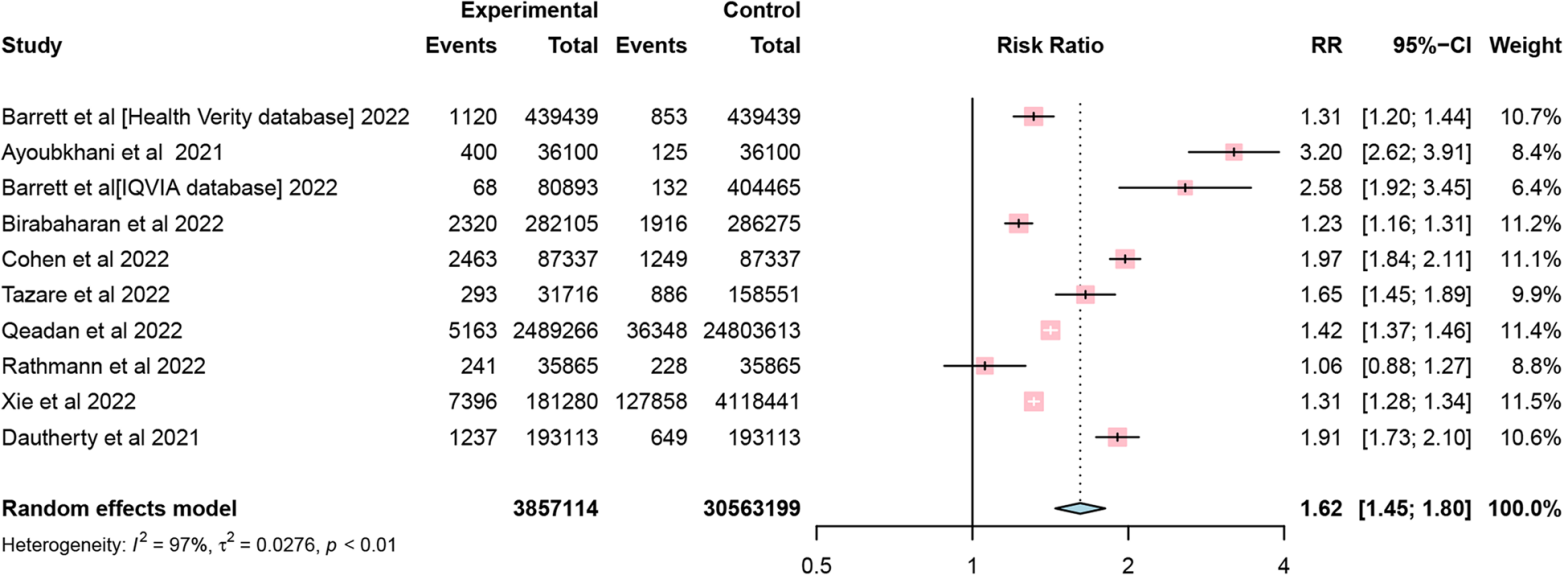
Forest plot comparing COVID-19 and non-COVID-19 for the outcome of diabetes. RR risk ratio, CI confidence interval

**Fig. 4:**
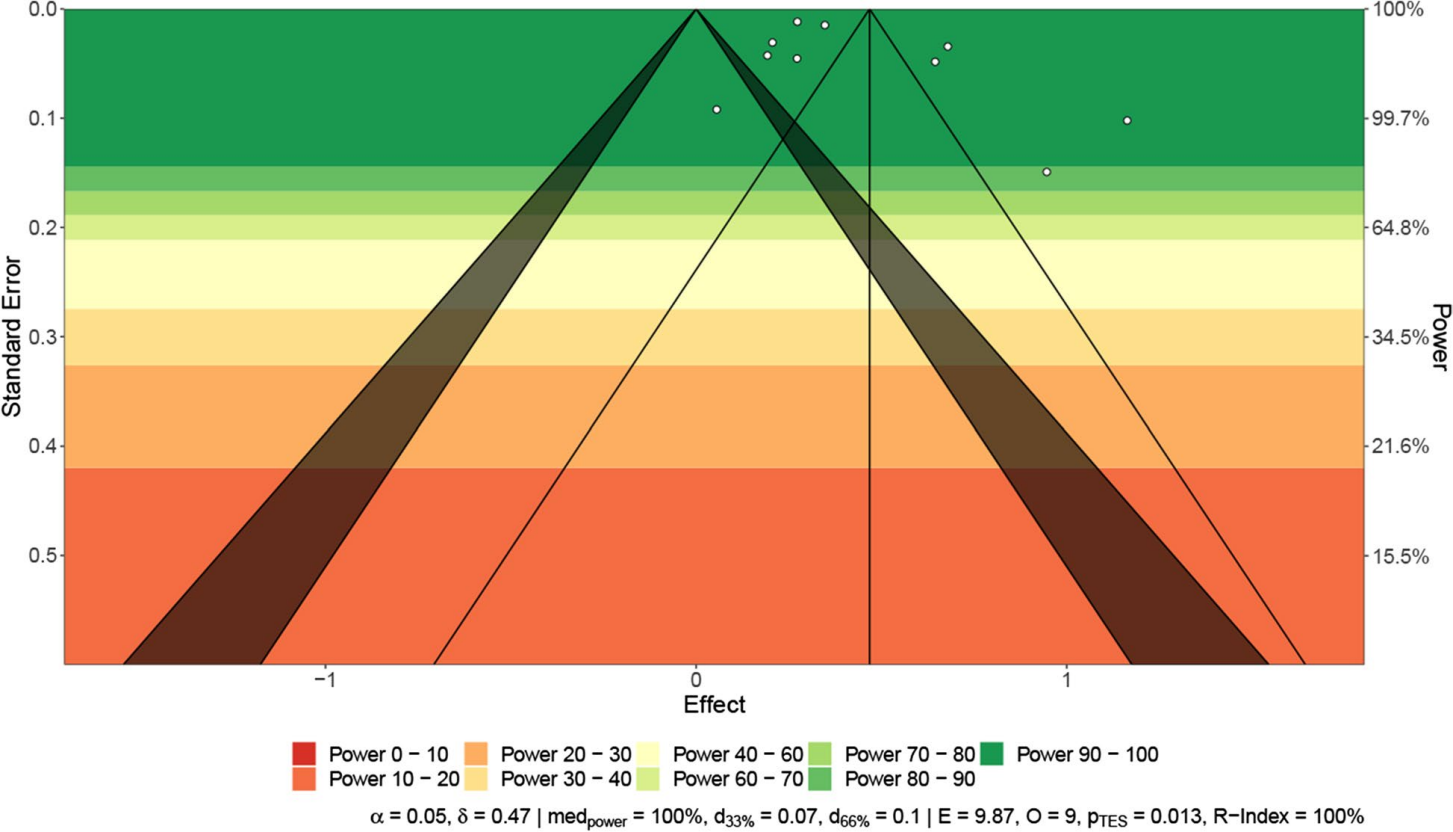
Sunset power-enhanced funnel plot for included studies. Egger’s test: *p* = 0.104. Different colors represent different ranges of statistical power. All studies included in the meta-analysis had statistical power greater than 90%

Of the nine included studies, although one study lacked specific follow-up times, the overall incidence of DM was found to be 15.53 cases per 1000 person-years of follow-up ([95% CI 7.91–25.64]; Fig. 2), using a random-effects meta-analysis. The relative risk of diabetes after COVID-19 was 1.62 ([95% CI 1.45–1.80]; Fig. 3) compared with patients not infected with COVID-19. Power-enhanced funnel plots (sunset funnel plot) and Egger’s test (*p* = 0.104) did not suggest publication bias (Fig. 4). In the sunset funnel plot, we found that all studies had strong statistical tests.

A subgroup analysis was performed looking at the type of new-onset diabetes. The risk of developing type 1 (insulin-dependent) diabetes was found by meta-analysis to be RR=1.48 ([95% CI 1.26–1.75]; Additional file 1: Fig. S1); type 2 (non-insulin-dependent) diabetes had a RR of 1.70 ([95% CI 1.32–2.19]; Additional file 1: Fig. S1); and those with an unspecified type of diabetes had a RR of 1.50 ([95% CI 0.87–2.58]; Additional file 1: Fig. S1) compared to the uninfected population.

Another subgroup analysis was performed according to whether the control group was or was not those with an upper respiratory tract infection. The relative risk of developing diabetes was increased 1.17-fold ([95% CI 1.02–1.34]; Additional file 1: Fig. S2) after COVID-19 compared to patients with upper respiratory tract infections. The relative risk of developing diabetes was increased 1.82-fold ([95% CI 1.47–2.24]; Additional file 1: Fig. S2) after COVID-19 compared to the general population.

Subgroup analysis was performed again according to the age of onset. The annual incidence rate per 1000 person-years of follow-up was 3.65 (95% CI, 2.91–4.83; Additional file 1: Fig. S3) in those <18 years; 15.53 ([95% CI 7.91–25.64]; Additional file 1: Fig. S3) in those ≥18 years; and 17.45 ([95% CI 16.77–18.14]; Additional file 1: Fig. S3) in those >65 years. At all ages, there was a statistically significant positive correlation between COVID-19 infection and the risk of developing diabetes. The positive correlation between COVID-19 infection and the risk of developing diabetes was statistically significant at all ages, <18 years: RR=1.72 ([95% CI 1.19–2.49]; Additional file 1: Fig. S4); ≥18 years: RR=1.63 ([95% CI 1.26–2.11]; Additional file 1: Fig. S4); >65 years: RR=1.68 ([95% CI 1.22–2.30]; Additional file 1: Fig. S4).

Finally, a subgroup analysis was performed according to gender. The incidence rate was 3.14 ([95% CI 0.75–7.15]; Additional file 1: Fig. S5) per 1000 person-years of follow-up in the male population and 3.00 ([95% CI 0.80–6.59]; Additional file 1: Fig. S5) per 1000 person-years of follow-up in the female population. The relative risk of developing diabetes was increased 2.08-fold in males ([95% CI 1.27–3.40]; Additional file 1: Fig. S6) and 2.15-fold in females ([95% CI 1.26–3.68]; Additional file 1: Fig. S6) after COVID-19 compared to a population without COVID-19 infection.

Follow-up time data were available for eight studies (with 9 cohorts) [29–31, 33–37]. The mean follow-up time of participants ranged from 64 to 352 days. The pooled cumulative incidence of diabetes reported at different follow-up time periods (Additional file 1: Fig. S7) was 2.19% (95% CI 1.36–3.21) at less than 3 months, 4.46% (95% CI 0.40–12.53) at 3 to 6 months, and 0.91% (95% CI 0.05–2.82) at more than 6 months. Furthermore, a significantly higher risk of new-onset diabetes was detected over the reported range of follow-up (Additional file 1: Fig. S8): RR 1.95 at less than 3 months (95% CI 1.85–2.06), RR 1.24 at 3 to 6 months (95% CI 1.12–1.37), and RR 1.38 at more than 6 months (5% CI 1.23–1.55).

Among the included studies, patients in six cohort studies [29–31, 34, 37] suffered from mild-to-moderate COVID-19; patients in three studies [32, 35, 36] included mild, moderate, and severe patients; only one study [33] did not parse the acuity levels of COVID-19 patients included. A subgroup analysis found that, compared with non-COVID-19 patients, the incidence of diabetes in the mild-to-moderate COVID-19 cohort was 2.96% ([95% CI 0.58–7.07]; Additional file 1: Fig. S9), and the risk was 1.48 times ([95% CI 1.25–1.75]; Additional file 1: Fig. S10); the incidence of diabetes in the severe patients was 11.65% ([95% CI 2.59–25.96]; Additional file 1: Fig. S9), and the risk was 1.67 times ([95% CI 1.25–2.23]; Additional file 1: Fig. S10).

**Fig. 5:**
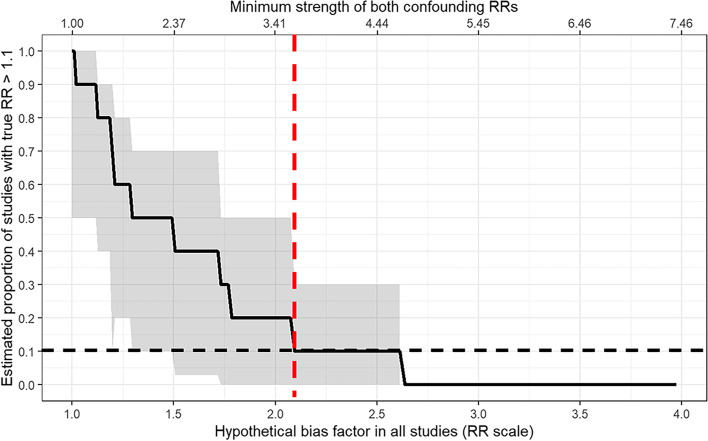
Sensitivity analysis for unmeasured confounding. RR risk ratio. The figure shows the potential impact of unmeasured confounding on the reported association of after-COVID-19 with diabetes mellitus. Specifically, it shows the change in the proportion of individual studies that would report a “true” association, defined as relative risk >1.1, between after-COVID-19 and diabetes mellitus under different scenarios

The sensitivity analysis for unmeasured confounding showed that to reduce the percentage of RR above 1.1 from 100 to 10%, we estimated a bias factor of at least 2.08 ([95% CI 1.55–2.84]; Fig. 5) would be required in each study. This means that unmeasured confounding would have needed to shift each study’s point estimate away from the null by 2.08-fold on the RR scale. Using the *E*-value metric, this bias factor is equivalent to unmeasured confounders in each study that affected both COVID-19 and diabetes by risk ratios of at least 3.58 each ([95% CI 2.43–5.12]; Additional file 1: Fig. S11 and Fig. S12).

## Discussion

This systematic review and meta-analysis provided comprehensive quantitative estimates of the incidence of diabetes in 10 post-COVID-19 populations. To our knowledge, this is the largest and most wide-ranging analysis of this kind to date. With nearly 40 million participants, and nearly 200,000 cases of diabetes reported, we found a post-COVID-19 incidence of diabetes per 1000 person-years of 15.53, and a relative risk of 1.62 compared to non-COVID-19-infected people. Subgroup analyses suggested that the risk of developing diabetes was also increased regardless of age, gender, type of diabetes, follow-up time, or level of COVID-19 severity, although undifferentiated diabetes did not have a significant relative risk. These results remained significant even after accounting for the possibility of unmeasured confounding.

Similar results have been reported in patients infected with other viruses, with an increased incidence of diabetes compared with those not infected [38–40]. Our subgroup analysis revealed a 1.2-fold increased risk of developing diabetes after COVID-19 compared to patients with other upper respiratory tract infections and a 1.82-fold increased risk of developing diabetes after COVID-19 compared to the general population. This reinforces the need for clinicians to pay attention to patients’ glucose metabolism in the post-acute phase of COVID-19.

There is also new evidence regarding the effect of the SARS-CoV-2 virus on pancreatic β-cell function [41]. It has been suggested that SARS-CoV-2 may affect the pancreas by acting on the mRNA of angiotensin-converting enzyme 2 (ACE2) in the endocrine and exocrine glands of the pancreas [11, 42]. The presence of SARS-CoV-2 antigen has recently been reported in the postmortem pancreas of patients who died from COVID-19 [43]. In addition, SARS-CoV-2 can induce a cytokine storm, an exaggerated immune response that produces a broad spectrum of cytokines, thereby establishing a systemic pro-inflammatory environment, which may play a role in promoting insulin resistance and β-cell hyperstimulation, ultimately leading to altered cellular function and the death of β-cells [44–46]. According to our subgroup analysis, there was a 1.48-fold increased risk of developing type 1 diabetes and a 1.7-fold increased risk of type 2 diabetes compared to patients not infected with COVID-19.

In our analysis, the incidence rate per 1000 person-years of follow-up was 3.65 (95% CI, 2.91 to 4.83), RR=1.72 (95% CI, 1.1 to 2.50) in the <18-year-old population, with similar results in adults and in those >65 years old. Moreover, the relative risk of morbidity was similar across genders. These findings underscore the importance of COVID-19 prevention in all age groups and genders, such as encouraging vaccination of all eligible children and adolescents [47].

Although all of the studies we included reduced confounders by adjusting for the risk of associated factors (propensity score matching) [48], concerns about possible bias due to uncontrolled confounders (e.g., comorbidities, socioeconomic environment, body mass index [BMI], etc.) remain [49]. Our study is the first meta-analysis to consider the *E*-value as a parameter of unmeasured confounders in examining the association between the COVID-19 post-acute phase and diabetes risk, which represents a new methodological contribution to the study of COVID-19 and diabetes [24]. The *E*-value, a sensitivity analysis of unmeasured confounders, is a relatively new method for measuring the association between exposure and outcome robustness and to assess evidence of causality [50]. Our results suggest that an unobserved confounder would need to be associated with a risk ratio of 2.08 for exposure and outcome to fully explain the mean RR of 1.62. In addition, a risk ratio of 3.58 would be required for the confounder to make the risk estimate statistically nonsignificant. Propensity matching was performed in all of the studies we included, and most studies adjusted for at least some clinically important confounding factors, such as patient age, gender, BMI, race, and comorbidities. Therefore, we believe it is implausible that residual confounders exist above and beyond these measured confounders that are sufficient to explain away the above results.

### Limitations

Several potential study limitations need to be considered. First, all included studies used a retrospective design and all studies used the breadth and depth of large electronic healthcare databases [29, 30, 32, 33, 35, 37, 51–53] to construct cohorts and define health characteristics based on validated definitions, which cannot exclude misclassification bias, particularly for diabetes types. Second, some of the studies used contemporary controls, not excluding the possibility that some individuals may be infected with SARS-CoV-2 and have not been tested, which could bias the results toward the null hypothesis if these individuals were present in large numbers in the contemporary control group. Third, because the included studies were conducted in different countries and in different regions within the same country, differences in national and regional care policies are expected, for which this meta-analysis could not be adapted or adjusted. Fourth, the study designs were heterogeneous (prospective cohort and retrospective cohort studies). As the number of available prospective cohort studies on this topic remains small, more high-quality studies are needed to confirm our results.

## Conclusions

Patients of all ages and genders recovering from COVID-19 had an elevated incidence and relative risk for developing diabetes. Particular attention should be paid to potential new-onset diabetes during the first 3 months of follow-up after COVID-19.

## Supplementary Information

**Additional file 1: Table S1.** Search strategies applied in each bibliographic database. **Table S2.** Characteristic of studies included in the present study for analyzing risk of incident diabetes post-COVID-19 versus matched controls. **Table S3.** Quality assessment of included studies with the Newcastle–Ottawa Scale. **Figure S1.** Subgroup analysis of diabetes type. **Figure S2.** Subgroup analysis to whether the control group was or was not upper respiratory tract infections. **Figure S3.** Forest plot showing the incidence of DM among different age groups after COVID-19. **Figure S4.** Subgroup analysis of different age groups. **Figure S5.** Forest plot showing the incidence of DM among different gender after COVID-19. **Figure S6.** Subgroup analysis of gender. **Figure S7.** Forest plot showing the incidence of diabetes among different follow-up time after COVID-19. **Figure S8.** Subgroup analysis of different follow-up time. **Figure S9.** Forest plot showing the incidence of diabetes based on different levels of COVID-19 severity. **Figure S10.** Subgroup analysis of different levels of COVID-19 severity. **Figure S11.** Bias factor = log(1). **Figure S12.** Bias factor = log(2.08).

**Additional file 2: Table S1.** PRISMA checklist. **Table S2.** MOOSE Statement - Reporting Checklist for Authors, Editors, and Reviewers of Meta-analyses of Observational Studies.

## Data Availability

All data were collected from publicly available literatures, and all data generated or analyzed during this study are included in this published article and its additional files.

## Abbreviations

ARDS: Acute respiratory distress syndrome
BMI: Body mass index
CI: Confidence interval
COVID-19: Coronavirus disease 2019
DM: Diabetes mellitus
*I*^2^: Heterogeneity measure
ICD: International Classification of Diseases
ICU: Intensive care unit
PSM: Propensity score matching
RR: Relative risk
SARS-CoV-2: Severe acute respiratory syndrome coronavirus 2
