# Supplementary material for: Risk for newly diagnosed diabetes after COVID-19: a systematic review and meta-analysis
Source: BMC Med. 2022 Nov 15;20:444. doi: 10.1186/s12916-022-02656-y (PMC9666960; doi:10.1186/s12916-022-02656-y)
Supplement: Supplementary file 1 — Additional file 1: Table S1. Search strategies applied in each bibliographic database. Table S2. Characteristic of studies included in the present study for analyzing risk of incident diabetes post-COVID-19 versus matched controls. Table S3. Quality assessment of included studies with the Newcastle–Ottawa Scale. Figure S1. Subgroup analysis of diabetes type. Figure S2. Subgroup analysis to whether the control group was or was not upper respiratory tract infections. Figure S3. Forest plot showing the incidence of DM among different age groups after COVID-19. Figure S4. Subgroup analysis of different age groups. Figure S5. Forest plot showing the incidence of DM among different gender after COVID-19. Figure S6. Subgroup analysis of gender. Figure S7. Forest plot showing the incidence of diabetes among different follow-up time after COVID-19. Figure S8. Subgroup analysis of different follow-up time. Figure S9. Forest plot showing the incidence of diabetes based on different levels of COVID-19 severity. Figure S10. Subgroup analysis of different levels of COVID-19 severity. Figure S11. Bias factor = log(1). Figure S12. Bias factor = log(2.08). [file 12916_2022_2656_MOESM1_ESM.docx]

# Additional File

# Title: Risk of incident diabetes in long COVID: A systematic review and meta-analysis

**contents**  **page**

[Additional File 1](#_Toc118487874)

[Title: Risk of incident diabetes in long COVID: A systematic review and meta-analysis 1](#_Toc118487875)

[Table S1. Search strategies applied in each bibliographic database. 4](#_Toc118487876)

[Table S2. Characteristic of studies included in the present study for analyzing risk of incident diabetes post-COVID-19 versus matched controls. 7](#_Toc118487896)

[Table S3. Quality assessment of included studies with the Newcastle–Ottawa Scale. 8](#_Toc118487897)

[Fig. S1. Subgroup analysis of diabetes type. 9](#_Toc118487898)

[Fig. S2. Subgroup analysis to whether the control group was or was not upper respiratory tract infectious. 9](#_Toc118487899)

[Fig. S3. Forest plot showing the incidence of DM among different age groups after COVID-19. 10](#_Toc118487900)

[Fig. S4. Subgroup analysis of different age groups. 10](#_Toc118487901)

[Fig. S5. Forest plot showing the incidence of DM among different gender after COVID-19. 11](#_Toc118487902)

[Fig. S6. Subgroup analysis of gender. 11](#_Toc118487903)

[Fig. S7. Forest plot showing the incidence of diabetes among different follow-up time after COVID-19. 12](#_Toc118487904)

[Fig. S8. Subgroup analysis of different follow-up time. 12](#_Toc118487905)

[Fig. S9. Forest plot showing the incidence of diabetes based on different levels of COVID-19 severity. 13](#_Toc118487906)

[Fig. S10. Subgroup analysis of different levels of COVID-19 severity. 13](#_Toc118487907)

[Fig. S11. Bias factor = log(1). 14](#_Toc118487908)

[Fig. S12 Bias factor = log(2.08). 14](#_Toc118487909)

Table S1. Search strategies applied in each bibliographic database.

| **Database** | **Search strategy** |
| --- | --- |
| PubMed | (((((((((((((COVID 19[Title/Abstract]) OR (SARS-CoV-2 Infection[Title/Abstract])) OR (Infection, SARS-CoV-2[Title/Abstract])) OR (SARS CoV 2 Infection[Title/Abstract])) OR (SARS-CoV-2 Infections[Title/Abstract])) OR (2019 Novel Coronavirus Disease[Title/Abstract])) OR (2019-nCoV Disease[Title/Abstract])) OR (2019-nCoV Disease[Title/Abstract])) OR (Infection, COVID-19 Virus[Title/Abstract])) OR (COVID-19[Title/Abstract])) OR (COVID-19 Pandemic[Title/Abstract])) OR (COVID-19 Pandemics[Title/Abstract])) AND (((((Diabetes Mellitus[Title/Abstract]) OR (Diabetes[Title/Abstract])) OR (DM[Title/Abstract])) OR (Diabetes Mellitus, Type 1[Title/Abstract])) OR (Diabetes Mellitus, Type 2[Title/Abstract]))) AND (((((((((cohort studies[Title/Abstract]) OR (longitudinal studies[Title/Abstract])) OR (follow-up studies[Title/Abstract])) OR (prospective studies[Title/Abstract])) OR (retrospective studies[Title/Abstract])) OR (cohort[Title/Abstract])) OR (longitudinal[Title/Abstract])) OR (prospective[Title/Abstract])) OR (retrospective[Title/Abstract])) |
| Embase | #4 #1 AND #2 AND #3  #3’cohort analysis’/exp OR ‘longitudinal study’/exp OR ‘prospective study’/exp OR ‘follow up’/exp OR cohort*:ab,ti  3,520,222  #2 ‘diabetes mellitus, type 2’:ab,ti OR ‘diabetes mellitus, type 1’:ab,ti OR ‘diabetes mellitus’:ab,ti OR ‘diabetes’:ab,ti  921,303  #1’covid-19’:ab,ti OR ‘sars-cov-2 infection’:ab,ti OR ‘infection, sars-cov-2’:ab,ti OR ‘sars cov 2 infection’:ab,ti OR ‘sars-cov-2 infections’:ab,ti OR ‘2019-ncov disease’:ab,ti OR ‘2019 ncov disease’:ab,ti OR ‘2019-ncov diseases’:ab,ti OR ‘disease, 2019-ncov’:ab,ti OR ‘covid-19 virus infection’:ab,ti OR ‘covid 19 virus infection’:ab,ti OR ‘covid-19 virus infections’:ab,ti OR ‘sars coronavirus 2 infection’:ab,ti OR ‘covid-19 virus disease’:ab,ti OR ‘2019-ncov infection’:ab,ti OR ‘2019 ncov infection’:ab,ti OR ‘2019-ncov infections’:ab,ti OR ‘infection, 2019-ncov’:ab,ti OR ‘covid19’:ab,ti OR ‘covid-19 pandemic’:ab,ti OR ‘covid 19 pandemic’:ab,ti OR ‘pandemic, covid-19’:ab,ti OR ‘covid-19 pandemics’:ab,ti |
| Web of Science | #1 TS=(COVID-19 OR SARS-CoV-2 Infection OR Infection, SARS-CoV-2 OR SARS CoV 2 Infection OR SARS-CoV-2 Infections OR 2019-nCoV Disease OR 2019 nCoV Disease OR 2019-nCoV Diseases OR Disease, 2019-nCoV OR COVID-19 Virus Infection OR COVID 19 Virus Infection OR COVID-19 Virus Infections OR SARS Coronavirus 2 Infection OR COVID-19 Virus Disease OR 2019-nCoV Infection OR 2019 nCoV  #2 **TS=(Diabetes Mellitus, Type 2 OR Diabetes Mellitus, Type 1 OR Diabetes Mellitus OR Diabetes)**  **#3** TS=(cohort studies OR longitudinal studies OR follow-up studies OR prospective studies OR retrospective studies OR cohort OR  longitudinal)  #4 **#1 AND #2 AND #3** |
| Cochrane Central Register of Controlled Trials | **#**1 (COVID-19):ab,ti,kw OR (SARS-CoV-2 Infection):ab,ti,kw OR (Infection, SARS-CoV-2):ab,ti,kw OR (SARS CoV 2 Infection):ab,ti,kw OR (SARS-CoV-2 Infections):ab,ti,kw OR (2019-nCoV Disease):ab,ti,kw OR (2019 nCoV Disease):ab,ti,kw OR (2019-nCoV Diseases):ab,ti,kw OR (Disease, 2019-nCoV):ab,ti,kw OR (COVID-19 Virus Infection):ab,ti,kw OR (COVID 19 Virus Infection):ab,ti,kw OR (COVID-19 Virus Infections):ab,ti,kw OR (SARS Coronavirus 2 Infection):ab,ti,kw OR (COVID-19 Virus Disease):ab,ti,kw OR (2019-nCoV Infection):ab,ti,kw OR (2019 nCoV Infection):ab,ti,kw OR (2019-nCoV Infections):ab,ti,kw  #2 (Diabetes Mellitus, Type 2):ab,ti,kw OR (Diabetes Mellitus, Type 1):ab,ti,kw OR (Diabetes Mellitus):ab,ti,kw OR (Diabetes):ab,ti,kw  **#3** (cohort studies):ab,ti,kw OR (longitudinal studies):ab,ti,kw OR (follow-up studies):ab,ti,kw OR (prospective studies):ab,ti,kw OR (retrospective studies):ab,ti,kw OR (cohort):ab,ti,kw OR ( longitudinal):ab,ti,kw  #4 **#1 AND #2 AND #3** |

Table S2. Characteristic of studies included in the present study for analyzing risk of incident diabetes post-COVID-19 versus matched controls.

| **First author(year)** | **Country** | **Study design** | **Data sources** | **Defination of COVID-19** | **Defination of Diabetes** | **Selection of COVID-19 population** | **Selection of controls** | **Propensity score matching** |
| --- | --- | --- | --- | --- | --- | --- | --- | --- |
| Xie, 2022 | USA | Mached cohort study | US Department of Veterans Affairs (VA) national health-care databases | ICD-10 | HbA1c > 6.4% or diabetes ICD10 codes | Participants having been COVID-19 positive between March 1, 2020- Sept 30, 2021 and were alive 30 days after COVID diagnosis, subsequently matched (n=181280) | VHA users from 2019 to march 1,2022 and not in COVID-19 group, matched for covariates (n=4118441) | PSM based on age, race, gender, race, BMI, area deprivation index , comorbidities (such as cancer, cardiovascular disease, cerebrovascular disease, chronic lung disease, dementia, HIV, hyperlipidemia, and peripheral artery disease) |
| Rathmann,2022 | Germany | Retrospective cohort study | The Disease Analyzer | ICD-10 | ICD-10 | Covid-19 first diagnosis between 1 March 2020 and 31 January 2021 and individuals with prescriptions of corticosteroids within 30 days after the index dates were excluded | AURI with index dates of first diagnosis between 1 March 2020 and 31 January 2021 and individuals with prescriptions of corticosteroids within 30 days after the index dates were excluded | PSM based on sex, age, health insurance coverage, index month and comorbidity (obesity, hypertension, hyperlipidemia, myocardial infarction, stroke) |
| Qeadan, 2022 | USA | Retrospective cohort study | Cerner Real-World Data | determined by diagnostic codes and lab results | ICD-10 | Patients eligible for analysis included those who had a confirmed diagnosis code of COVID-19 or a positive SARS-CoV-2 lab result at encounters from December 1, 2019 through July 31, 2021, of type: “emergency”, “inpatient”, “admitted for observation”, “inpatient hospice care”, or “urgent care.” | patients treated in the same health systems as these COVID-19 patients, seen at least once since January 1, 2019 and until July 31, 2021 | Not applicable |
| Collaborative, 2021 | England | Retrospective cohort study | Electronic health record | ICD-10 | ICD-10 | All patients ≥18 years who hospitalised with COVID-19 in 2020 | A general population frequency matched cohort in 2019 | PSM based on age, sex and region |
| Cohen, 2022 | USA | Retrospective cohort study | two data sources within the UnitedHealth Group Clinical Research Database | ICD-10 or report of positive SARS-CoV-2polymerase chain reaction(PCR) | ICD-10 | Individuals were aged ≥65 years in 2020 and had: a primary, secondary, or tertiary diagnosis of covid-19 | Individuals were enrolled in Medicare Advantage with coverage of prescription drugs, were aged ≥65 years in 2020, and did not have a clinical diagnosis related to covid-19 or a positive PCR test in 2020 | Matching by propensity score to create three comparison groups that were similar to the SARS-CoV-2 group in terms of baseline characteristics and relevant confounders and based on 120 variables by logistic regression with ridge penalty |
| Birabaharan, 2022 | USA | Retrospective cohort study | TriNetx Analytics Network | ICD-10 or report of positive SARS-CoV-2polymerase chain reaction(PCR) | ICD-10 | People diagnosed from 20 January 2020 to 20 January 2021 with COVID-19 and individuals with prescriptions of corticosteroids within 30 days after the index dates were excluded | people diagnosed with influenza (ICD-10 J09-J11) from 20 January 2018 to 20 January 2021 and individuals with prescriptions of corticosteroids within 30 days after the index dates were excluded | PSM based on age, sex, race, ethnicity, obesity, hypertension, hyperlipidemia, nicotine dependence, substance use, socioeconomic deprivation, and family history of diabetes |
| Barrett [IQVIA database], 2022 | USA | Retrospective cohort study | IQVIA PharMetrics Plus | ICD-10 | one or more health care claims with a diabetes diagnosis (ICD-10) occurring >30 days after the index date | Patients aged <18 years with diag­nosed COVID-19 from IQVIA health care claims data (March 1, 2020- February 26, 2021) | Subjects who did not receive a COVID-19 diagnosis during pandemic, matched by age and sex | PSM based on age, sex |
| Barrett [Health Verity database], 2022 | USA | Retrospective cohort study | HealthVerity | ICD-10 diagnosis code for COVID-19 or a positive SARS-CoV-2 polymerase chain reaction(PCR) | one or more health care claims with a diabetes diagnosis (ICD-10) occurring >30 days after the index date | Patients <18 years who had any health care encounter possibly related to COVID-19 | Subjects without COVID-19 diagnosis during pandemic, matched by age and sex | PSM based on age, sex |
| Ayoubkhani, 2021 | England | Retrospective cohort study | Hospital Episode Statistics Admitted Patient Care & General Practice Extraction Service Data for Pandemic Planning and Research (GDPPR) | ICD-10 | DM (ICD-10 codes) outcomes ascertained from index date (date of discharge from first hospitalisation for COVID-19) | Individuals were included if they had a hospital episode from 1 January to 31 August 2020 with a primary diagnosis of covid-19 | Individuals in general population without COVID-19 diagnosis; having one GDPPR record between 1 January 2019 (one year before the start of the follow-up period) and 30 September 2020 | PSM based on age, sex, ethnicity, region, and deprivation and comorbidities (such as cancer, hypertension, BMI, smoking status) |
| Dautherty, 2021 | USA | Retrospective cohort study | UnitedHealth Group Clinical Discovery Database | ICD-10 | DM (ICD-10 codes) outcomes ascertained from COVID-19 diagnosis plus 14 days or 21 days or 28 days (this cut-off based data selected for present study) | Patients aged 18-65 enrolled in health plan (1 January, 2019-April 1,2020) having documented diagnosis of COVID-19 or related healthcare claims upto October 31,2020 | Individuals aged 18-65 years without COVID-19 from 1 January 2019 to a randomly assigned index date drawn from COVID-19 group (2020 comparator group) | PSM based on age, sex, race, socioeconomic status to zip codes, pre-existing comorbidities, total length of stay as an inpatient, previous number of visits to a primary care physician, cardiologist, or nephrologist |
| AURI: acute upper respiratory tract infections; PSM: Propensity score matching; BMI: body mass index | | | | | | | | |

Table S3. Quality assessment of included studies with the Newcastle–Ottawa Scale.

| **Study name** | **Selection** | **Comparability** | **Outcome** | **Overall score** |
| --- | --- | --- | --- | --- |
| Xie et al. | **** | ** | *** | 9/9 |
| Rathmann et al. | **** | ** | *** | 9/ 9 |
| Qeadan et al. | **** | ** | * | 7/ 9 |
| Collaborative et al. | **** | ** | *** | 9/ 9 |
| Cohen et al. | **** | ** | *** | 9/ 9 |
| Birabaharan et al. | **** | ** | *** | 9/ 9 |
| Barrett et al.^a^ | **** | ** | *** | 9/ 9 |
| Barrett et al.^b^ | **** | ** | *** | 9/ 9 |
| Ayoubkhani | **** | ** | *** | 9/ 9 |
| Dautherty | **** | ** | *** | 9/ 9 |
| **Overall Score** | **40/ 40** | **20/20** | **28/30** | **88/ 90** |

Barrett et al.^a^: Barrett [IQVIA database]; Barrett et al.^b^: Barrett [Health Verity database].

Fig. S1. Subgroup analysis of diabetes type.


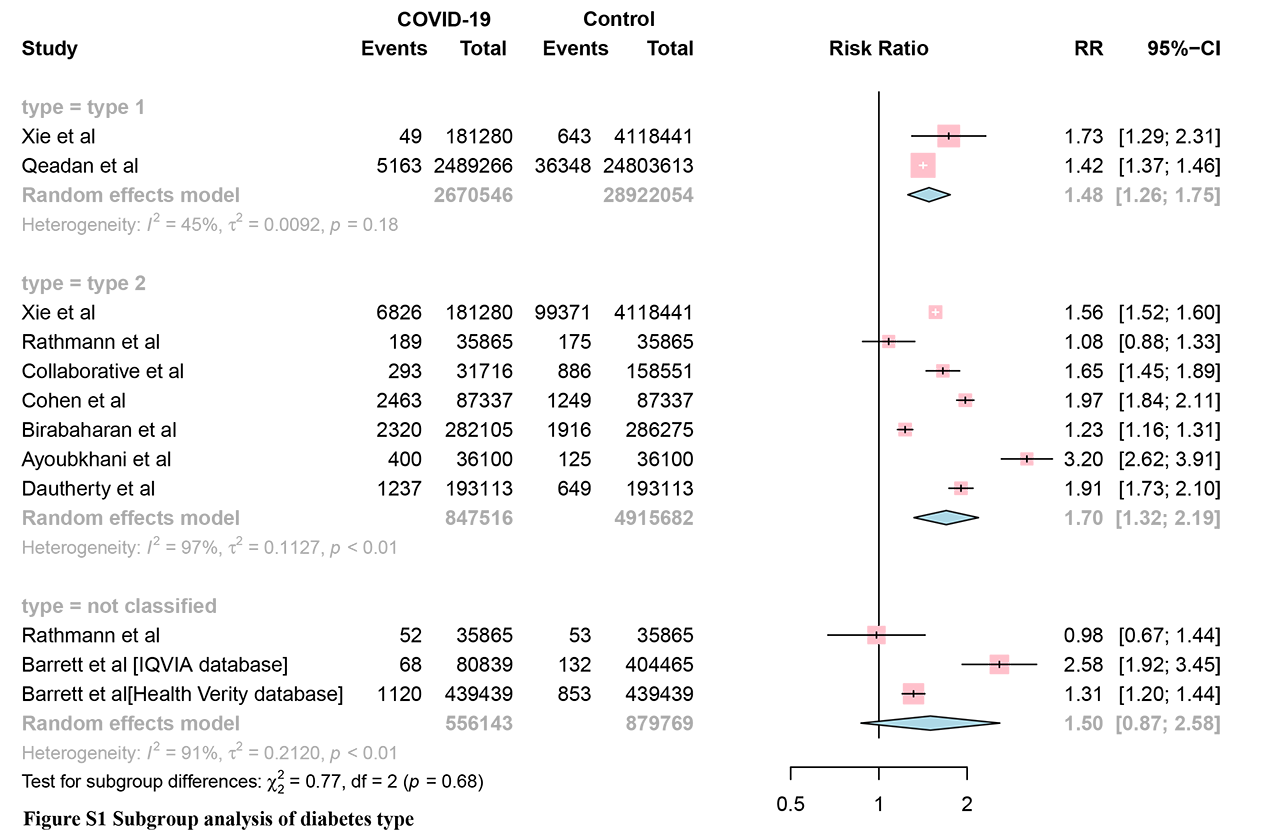


Fig. S2. Subgroup analysis to whether the control group was or was not upper respiratory tract infectious.


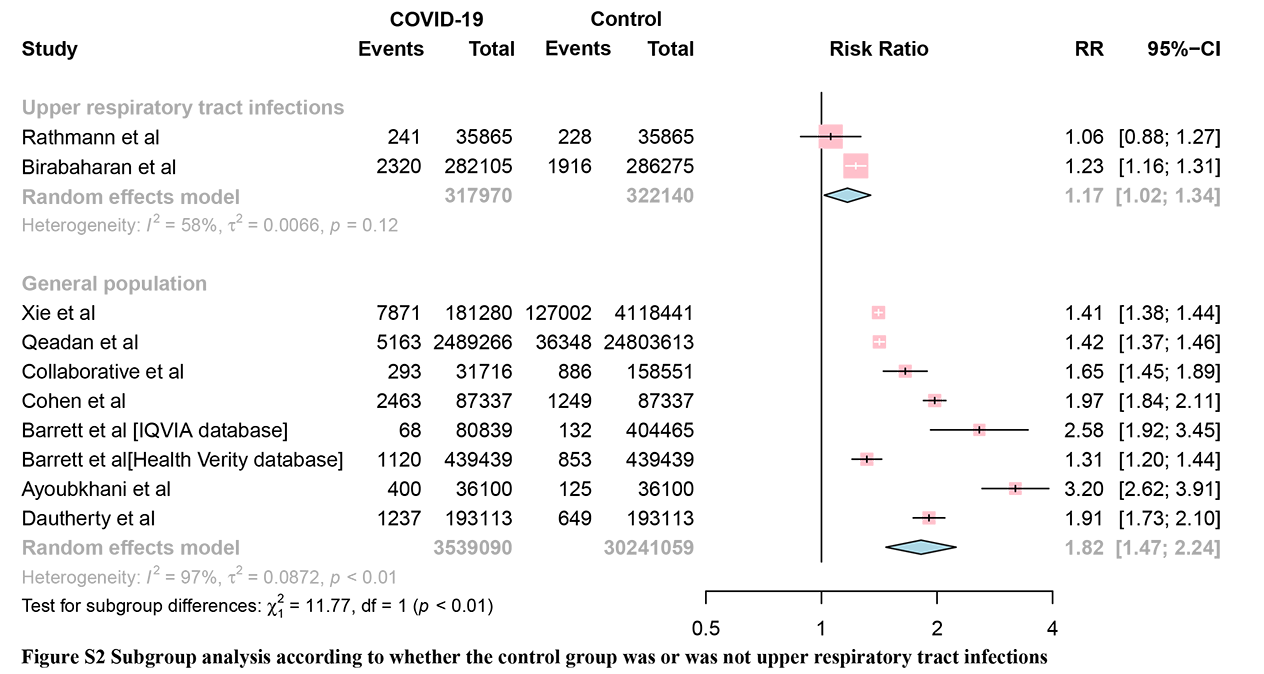


Fig. S3. Forest plot showing the incidence of DM among different age groups after COVID-19.


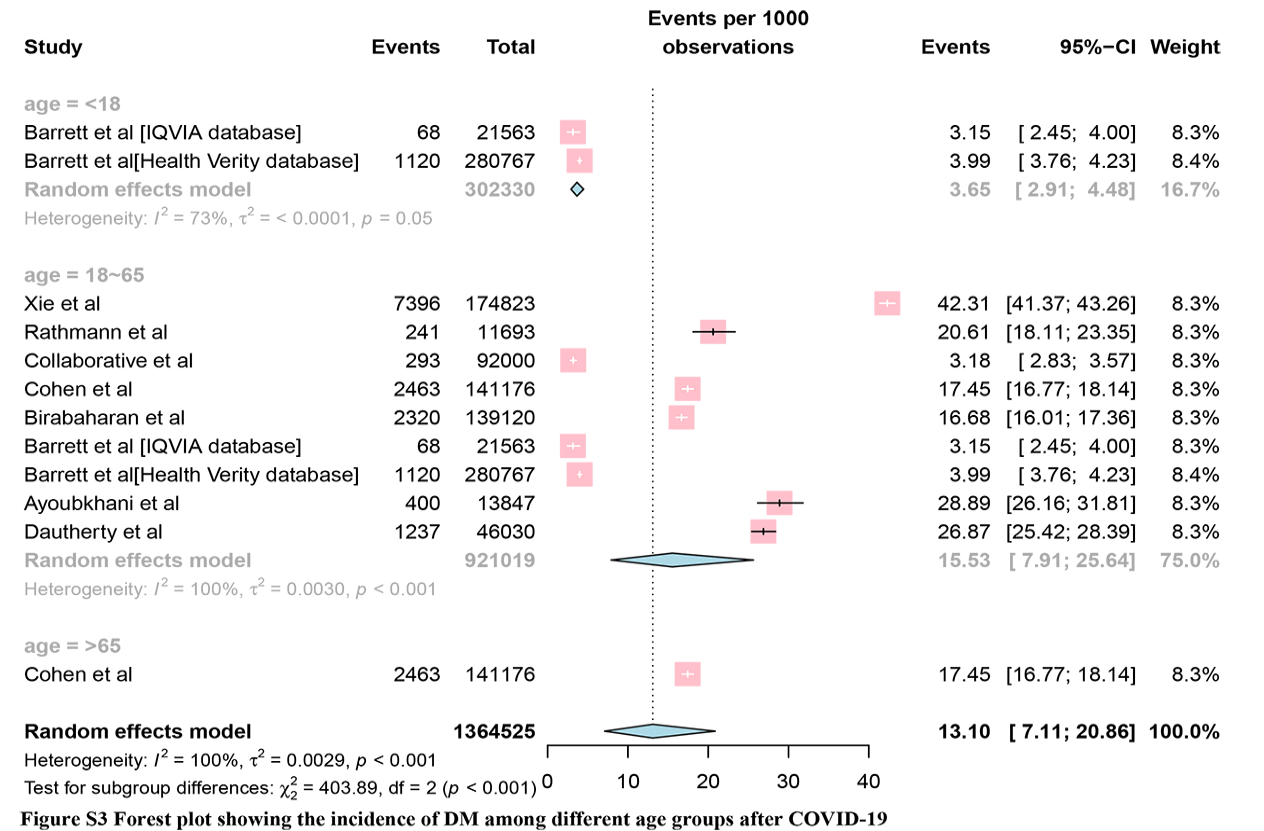


Fig. S4. Subgroup analysis of different age groups.


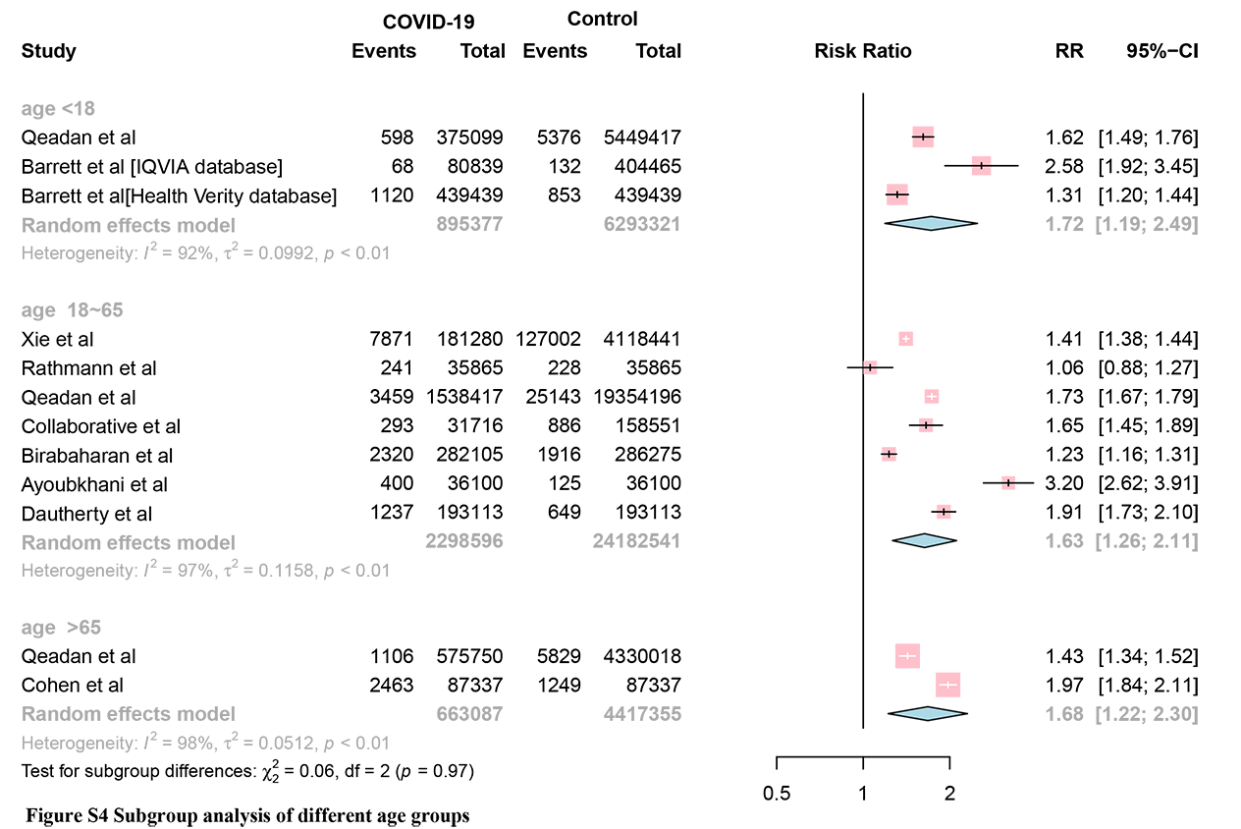


Fig. S5. Forest plot showing the incidence of DM among different gender after COVID-19.


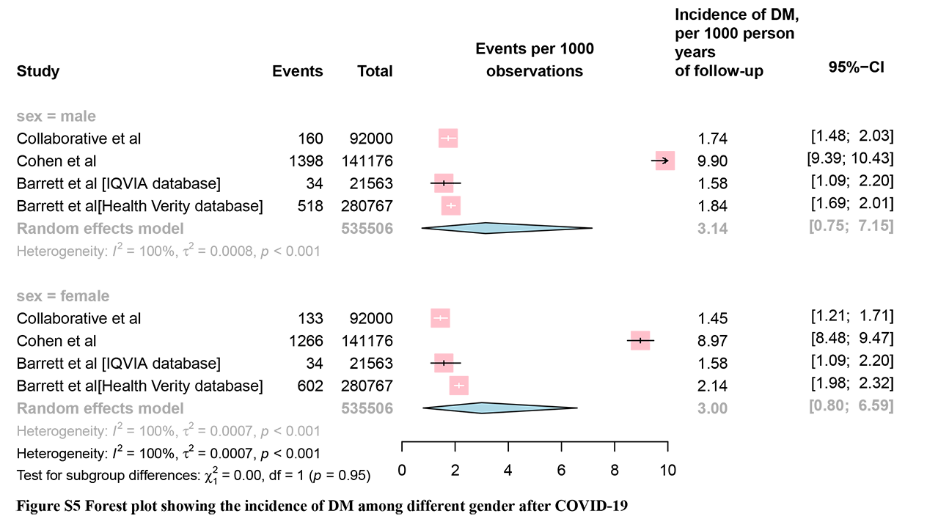


Fig. S6. Subgroup analysis of gender.


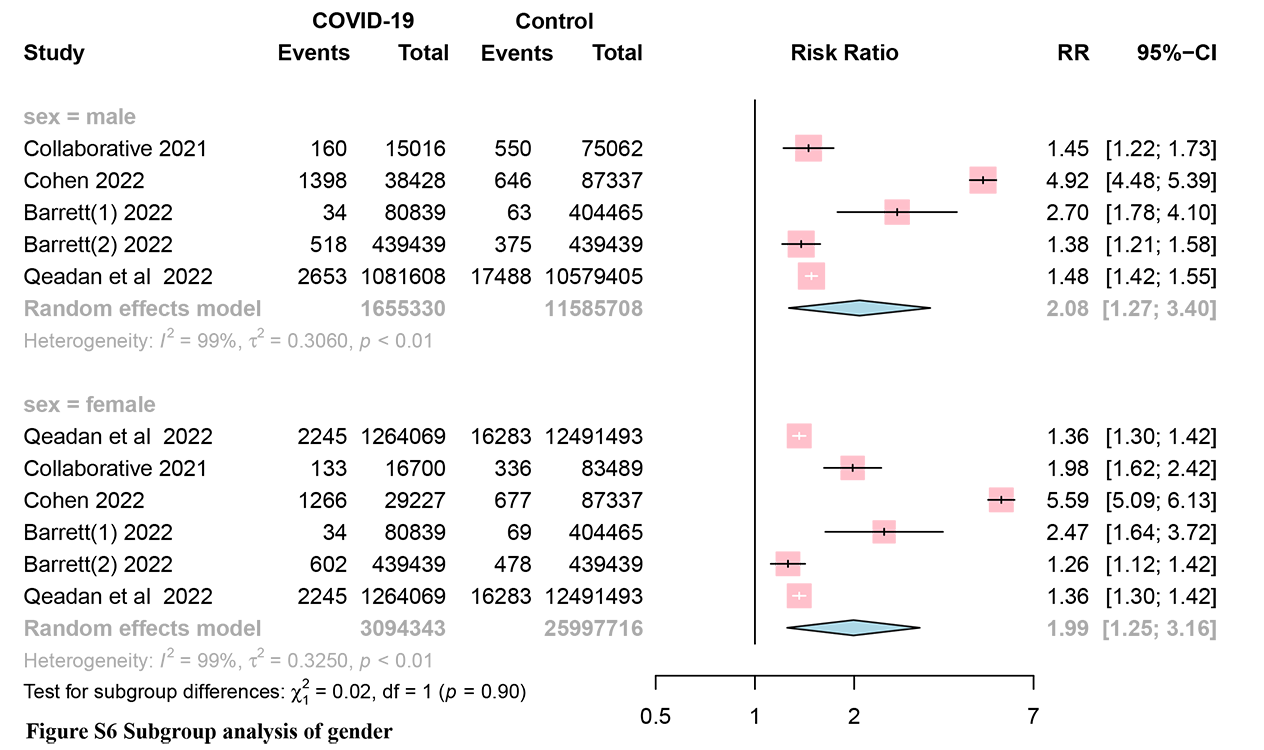


Fig. S7. Forest plot showing the incidence of diabetes among different follow-up time after COVID-19.


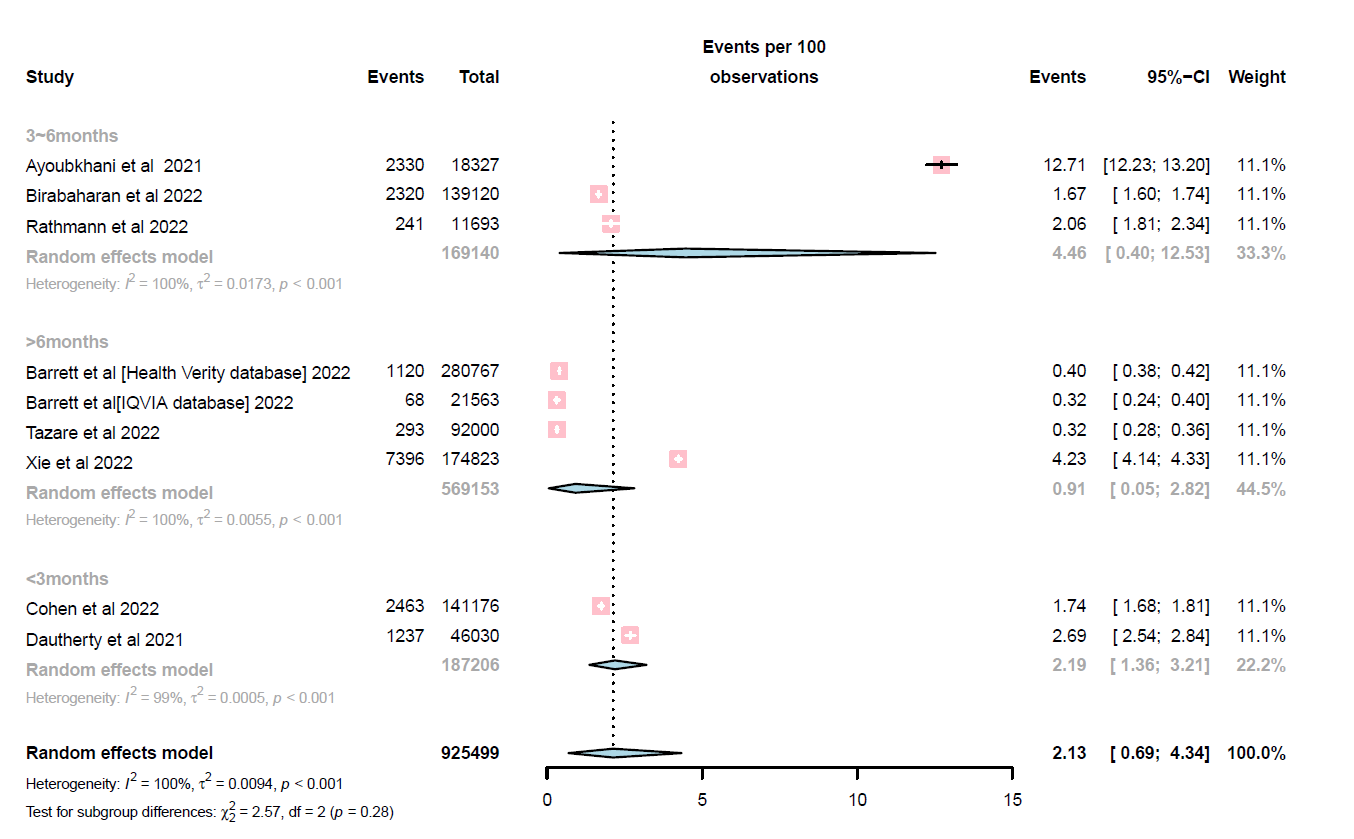


Fig. S8. Subgroup analysis of different follow-up time.


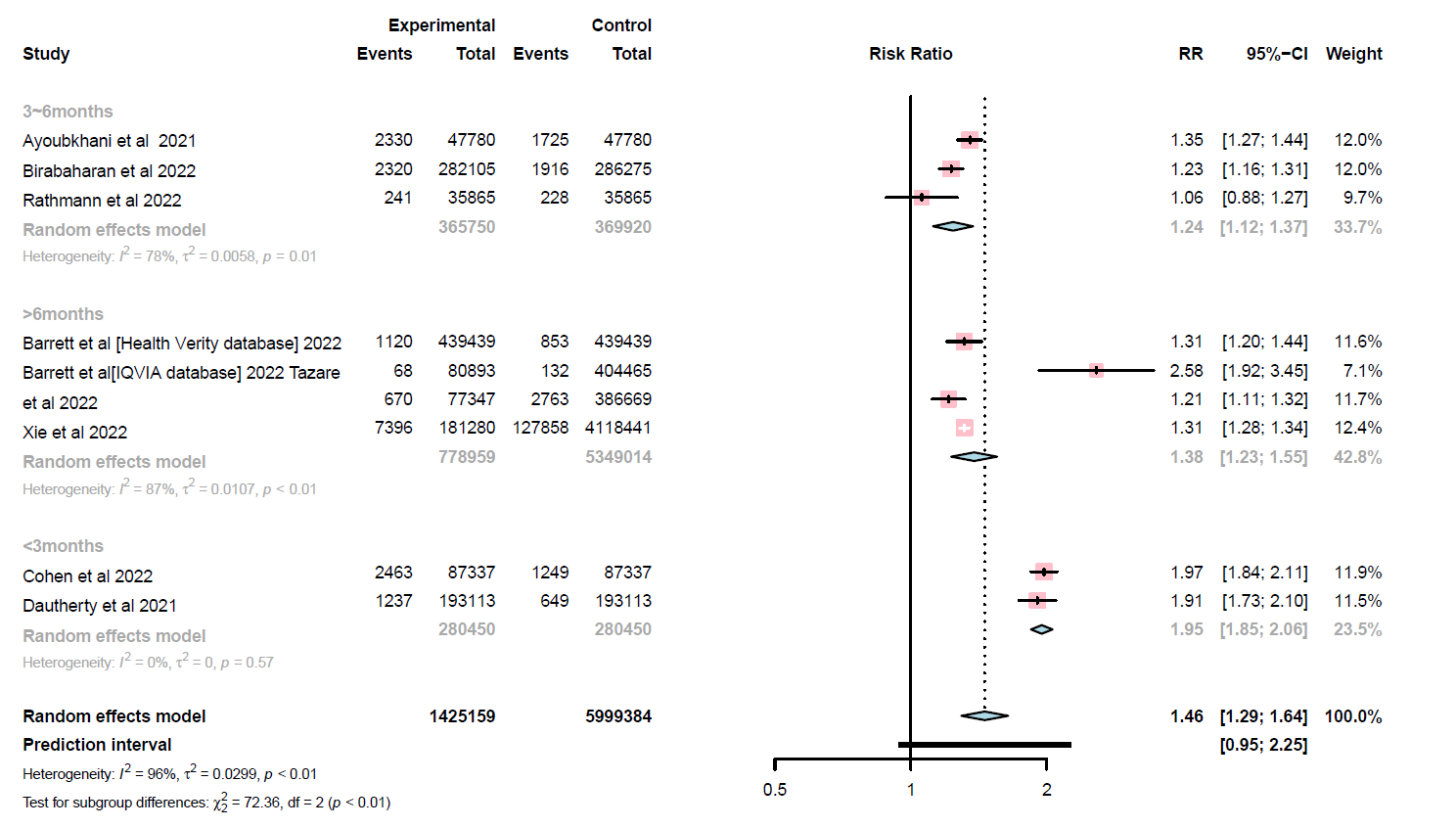


Fig. S9. Forest plot showing the incidence of diabetes based on different levels of COVID-19 severity.


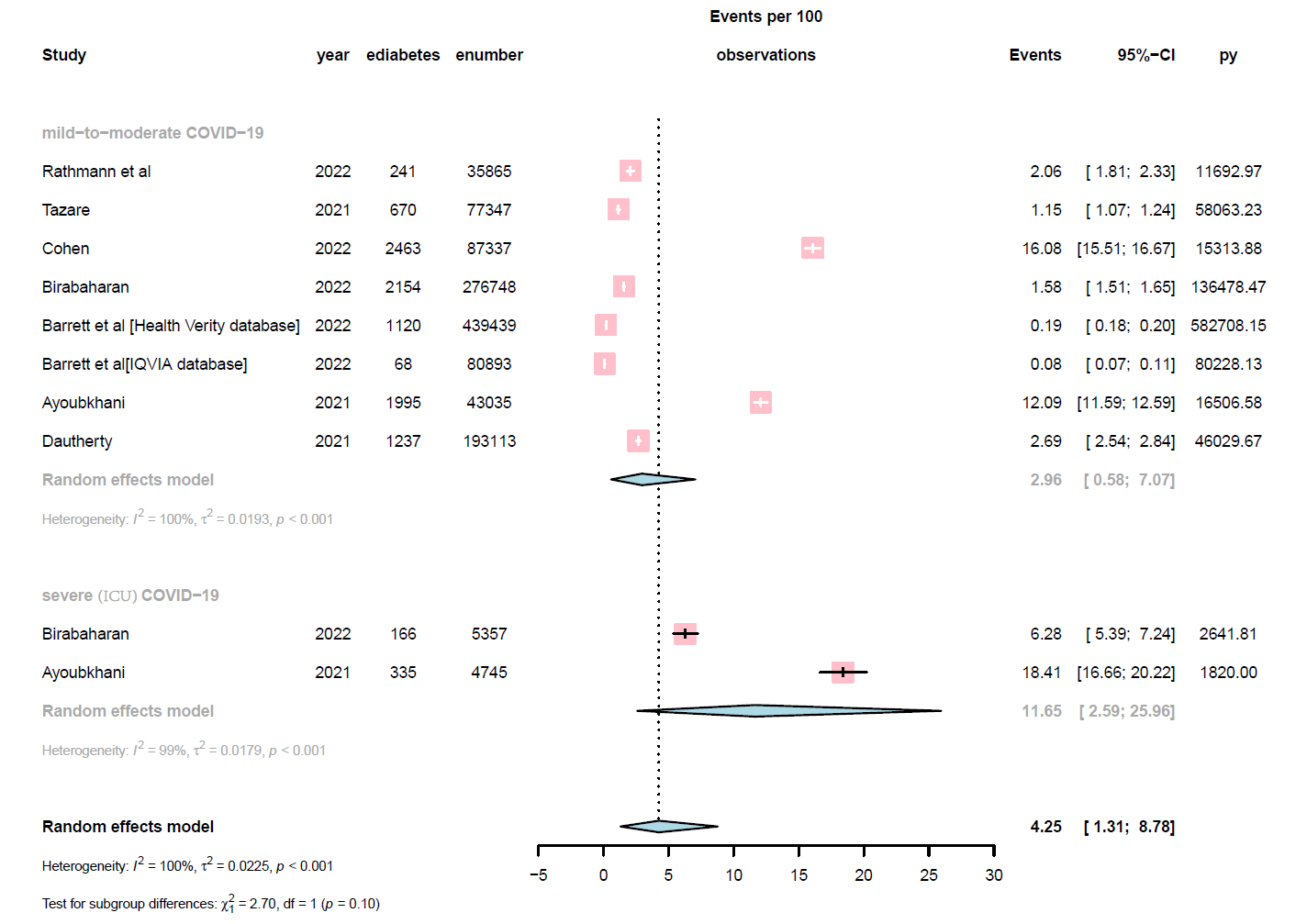


Fig. S10. Subgroup analysis of different levels of COVID-19 severity.


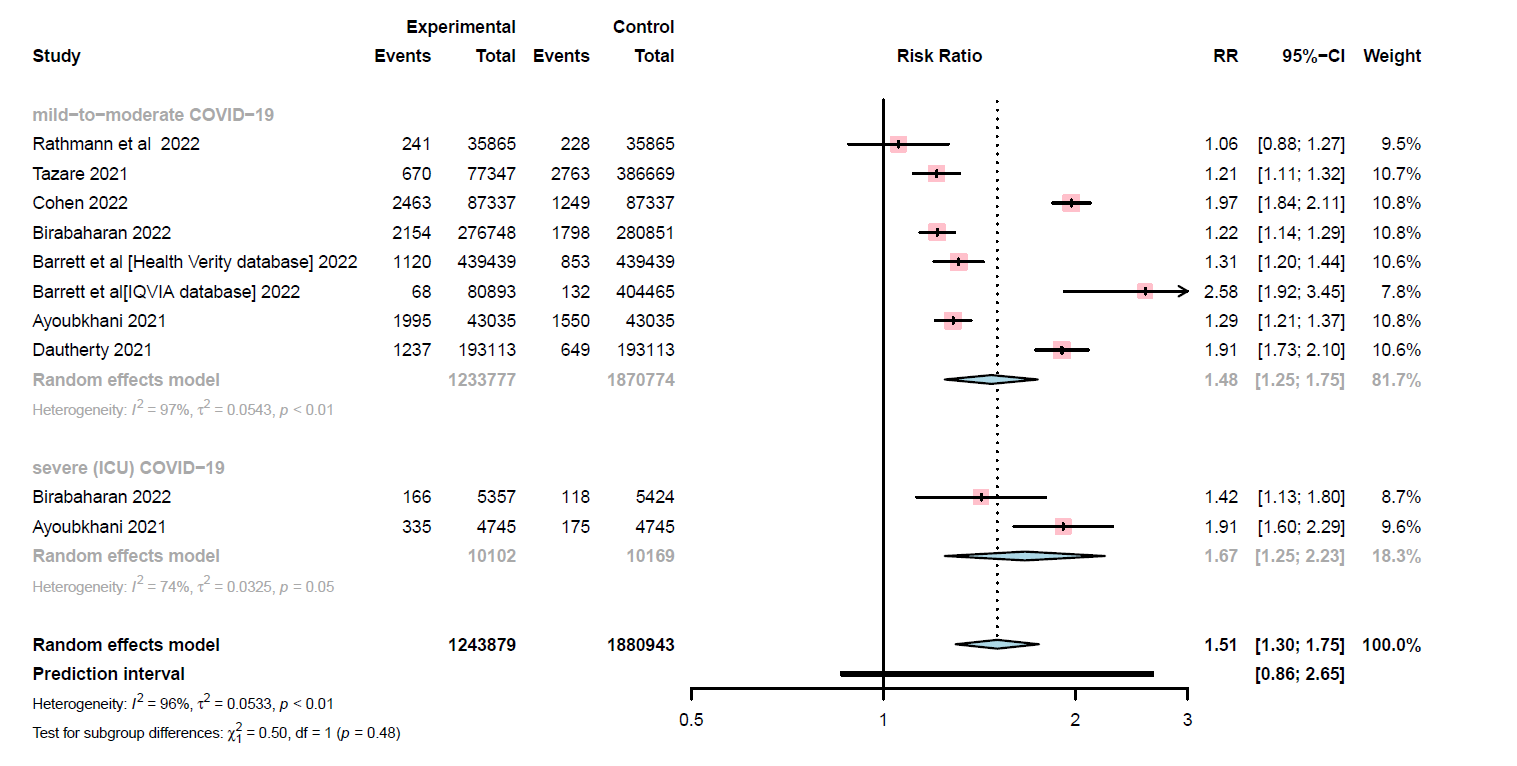


Fig. S11. Bias factor = log(1).





Fig. S12 Bias factor = log(2.08).




Fig. S11 and Fig. S12 show the potential impact of unmeasured confounding on the reported association of after COVID-19 with diabetes mellitus (DM). Specifically, it shows the change in the proportion of individual studies that would report a “true” association, defined as relative risk >1.1, between after COVID-19 and DM under different scenarios.
