# Supplementary material for: Risk for newly diagnosed diabetes after COVID-19: a systematic review and meta-analysis
Source: BMC Med. 2022 Nov 15;20:444. doi: 10.1186/s12916-022-02656-y (PMC9666960; doi:10.1186/s12916-022-02656-y)
Supplement: Supplementary file 2 — Additional file 2: Table S1. PRISMA checklist. Table S2. MOOSE Statement - Reporting Checklist for Authors, Editors, and Reviewers of Meta-analyses of Observational Studies. [file 12916_2022_2656_MOESM2_ESM.docx]

Table S1. PRISMA checklist.

| **Section/topic** | **#** | **Checklist item** | **Reported**  **on page #** |
| --- | --- | --- | --- |
| **TITLE** | | |  |
| Title | 1 | Risk for newly diagnosed diabetes after COVID-19: A systematic review and meta-analysis | 1 |
| **ABSTRACT** | | |  |
| Structured summary | 2 | Provide a structured summary including, as applicable: background; objectives; data sources; study eligibility criteria, participants, and interventions; study appraisal and synthesis methods; results; limitations; conclusions and implications of key findings; systematic review registration number. | 4-6 |
| **INTRODUCTION** | | |  |
| Rationale | 3 | Describe the rationale for the review in the context of what is already known. | 6 |
| Objectives | 4 | Provide an explicit statement of questions being addressed with reference to participants, interventions, comparisons, outcomes, and study design (PICOS). | 7 |
| **METHODS** | | |  |
| Protocol and registration | 5 | Indicate if a review protocol exists, if and where it can be accessed (e.g., Web address), and, if available, provide registration information including registration number. | 7-11 |
| Eligibility criteria | 6 | Specify study characteristics (e.g., PICOS, length of follow-up) and report characteristics (e.g., years considered, language, publication status) used as criteria for eligibility, giving rationale. | 7-8 |
| Information sources | 7 | Describe all information sources (e.g., databases with dates of coverage, contact with study authors to identify additional studies) in the search and date last searched. | 8 |
| Search | 8 | Present full electronic search strategy for at least one database, including any limits used, such that it could be repeated. | 8,Table S1 |
| Study selection | 9 | State the process for selecting studies (i.e., screening, eligibility, included in systematic review, and, if applicable, included in the meta-analysis). | 8, figure 1 |
| Data collection process | 10 | Describe method of data extraction from reports (e.g., piloted forms, independently, in duplicate) and any processes for obtaining and confirming data from investigators. | 8 |
| Data items | 11 | List and define all variables for which data were sought (e.g., PICOS, funding sources) and any assumptions and simplifications made. | 8 |
| Risk of bias in individual studies | 12 | Describe methods used for assessing risk of bias of individual studies (including specification of whether this was done at the study or outcome level), and how this information is to be used in any data synthesis. | 9 |
| Summary measures | 13 | State the principal summary measures (e.g., risk ratio, difference in means). | 9 |
| Synthesis of results | 14 | Describe the methods of handling data and combining results of studies, if done, including measures of consistency (e.g., I^2^) for each meta-analysis. | 9 |

| **Section/topic** | **#** | **Checklist item** | **Reported on**  **page #** |
| --- | --- | --- | --- |
| Risk of bias across studies | 15 | Specify any assessment of risk of bias that may affect the cumulative evidence (e.g., publication bias, selective reporting within studies). | 9 |
| Additional analyses | 16 | Describe methods of additional analyses (e.g., sensitivity or subgroup analyses, meta-regression), if done, indicating which were pre-specified. | 9-10 |
| **RESULTS** | | |  |
| Study selection | 17 | Give numbers of studies screened, assessed for eligibility, and included in the review, with reasons for exclusions at each stage, ideally with a flow diagram. | 10, Figure 1 |
| Study characteristics | 18 | For each study, present characteristics for which data were extracted (e.g., study size, PICOS, follow-up period) and provide the citations. | 10 Table 1 |
| Risk of bias within studies | 19 | Present data on risk of bias of each study and, if available, any outcome level assessment (see item 12). | 11, Table S3 |
| Results of individual studies | 20 | For all outcomes considered (benefits or harms), present, for each study: (a) simple summary data for each intervention group (b) effect estimates and confidence intervals, ideally with a forest plot. | Figure2, figure3, Table 1 and Table S2 |
| Synthesis of results | 21 | Present results of each meta-analysis done, including confidence intervals and measures of consistency. | 12-15 |
| Risk of bias across studies | 22 | Present results of any assessment of risk of bias across studies (see Item 15). | N/A |
| Additional analysis | 23 | Give results of additional analyses, if done (e.g., sensitivity or subgroup analyses, meta-regression [see Item 16]). | 15, Figure 4, Figure7, Figure 8 |
| **DISCUSSION** | | |  |
| Summary of evidence | 24 | Summarize the main findings including the strength of evidence for each main outcome; consider their relevance to key groups (e.g., healthcare providers, users, and policy makers). | 16-19 |
| Limitations | 25 | Discuss limitations at study and outcome level (e.g., risk of bias), and at review-level (e.g., incomplete retrieval of identified research, reporting bias). | 18-19 |
| Conclusions | 26 | Provide a general interpretation of the results in the context of other evidence, and implications for future research. | 19 |
| **FUNDING** | | |  |
| Funding | 27 | Describe sources of funding for the systematic review and other support (e.g., supply of data); role of funders for the systematic review. | 27 |

Table S2. MOOSE Statement - Reporting Checklist for Authors, Editors, and Reviewers of Meta-analyses of Observational Studies.

| **Reporting Criteria** | **Reported (Yes/No)** | **Reported on Page** |
| --- | --- | --- |
| **Reporting of Background** |  |  |
| Problem definition | Yes | 6 |
| Hypothesis statement | N/A | -- |
| Description of Study Outcome(s) | Yes | 12-16 |
| Type of exposure or intervention used | N/A | -- |
| Type of study design used | Yes | 7 |
| Study population | Yes | 8 |
| **Reporting of Search Strategy** |  |  |
| Qualifications of searchers (eg, librarians and investigators) | No | -- |
| Search strategy, including time period included in the synthesis and keywords | Yes | 8-9 |
| Effort to include all available studies, including contact with authors | Yes | 8 |
| Databases and registries searched | Yes | 8-9 |
| Search software used, name and version, including special features used (eg, explosion) | Yes | 8-9 |
| Use of hand searching (eg, reference lists of obtained articles) | Yes | 10 |
| List of citations located and those excluded, including justification | Yes | 8-10, Figure 1 |
| Method for addressing articles published in languages other than English | N/A | -- |
| Method of handling abstracts and unpublished studies | N/A | -- |
| Description of any contact with authors | N/A | -- |
| **Reporting of Methods** |  |  |
| Description of relevance or appropriateness of studies assembled for assessing the hypothesis to be tested | Yes | 10-11,Table1 and Table S2 |
| Rationale for the selection and coding of data (eg, sound clinical principles or convenience) | Yes | Figure 1 |
| Documentation of how data were classified and coded (eg, multiple raters, blinding, and interrater reliability) | Yes | 8-9 |
| Assessment of confounding (eg, comparability of cases and controls in studies where appropriate | Yes | 15-16, Fig. 5, Fig. S11,Fig. S12. |
| Assessment of study quality, including blinding of quality assessors; stratification or regression on possible predictors of study results | Yes | 11 |
| Assessment of heterogeneity | N/A | -- |
| Description of statistical methods (eg, complete description of fixed or random effects models, justification of whether the chosen models account for predictors of study results, dose-response models, or cumulative meta-analysis) in sufficient detail to be replicated | Yes | 9 |
| Provision of appropriate tables and graphics | Yes | 10-13 |
| **Reporting of Results** |  |  |
| Table giving descriptive information for each study included | Yes | 12, Table |
| Results of sensitivity testing (eg, subgroup analysis) | Yes | 13-15 |
| Indication of statistical uncertainty of findings | N/A | -- |
| **Reporting of Discussion** |  |  |
| Quantitative assessment of bias (eg, publication bias) | Yes | 12 |
| Justification for exclusion (eg, exclusion of non–English-language citations) | N/A | -- |
| Assessment of quality of included studies | Yes | 12-13 |
| **Reporting of Conclusions** |  |  |
| Consideration of alternative explanations for observed results | Yes | 16-18 |
| Generalization of the conclusions (ie, appropriate for the data presented and within the domain of the literature review) | Yes | 19 |
| Guidelines for future research | Yes | 16-19 |
| Disclosure of funding source | Yes | 27 |
